# Understanding females with attention deficit and hyperactivity disorder

**DOI:** 10.1192/j.eurpsy.2024.127

**Published:** 2024-08-27

**Authors:** O. Kilic

**Affiliations:** Department of Neurosciences, Bezmialem Vakif University, Institute of Health Sciences, Istanbul, Türkiye

## Abstract

**Abstract:**

The predominant association of attention deficit and hyperactivity disorder (ADHD) with males, often leads to underdiagnosis or misdiagnosis in females. Recent studies have highlighted marked differences between genders in the manifestation, symptoms, and outcomes of ADHD. Understanding these differences is essential for accurate identification, diagnosis, and tailored interventions for affected individuals, particularly females. TThe multifaceted nature of ADHD demands a nuanced examination of its impact on females, considering how societal expectations, hormonal influences, and a range of symptom presentations may contribute to the variation in the manifestation of this disorder across genders. This presentation aims to contribute to a more comprehensive understanding of ADHD, fostering improved recognition and tailored strategies to support both males and females who suffer from this condition.

**Disclosure of Interest:**

None Declared

